# Annexin A1 Preferentially Predicts Poor Prognosis of Basal-Like Breast Cancer Patients by Activating mTOR-S6 Signaling

**DOI:** 10.1371/journal.pone.0127678

**Published:** 2015-05-22

**Authors:** Anjana Bhardwaj, Nivetha Ganesan, Kazunoshin Tachibana, Kimal Rajapakshe, Constance T. Albarracin, Preethi H. Gunaratne, Cristian Coarfa, Isabelle Bedrosian

**Affiliations:** 1 Department of Surgical Oncology, The University of Texas MD Anderson Cancer Center, Houston, TX, United States of America; 2 Department of Molecular and Cellular Biology, Baylor College of Medicine, Houston, TX, United States of America; 3 Department of Pathology, The University of Texas MD Anderson Cancer Center, Houston, TX, United States of America; 4 Department of Biology and Biochemistry, University of Houston, Houston, TX, United States of America; University of South Alabama, UNITED STATES

## Abstract

**Introduction:**

Annexin A1 (ANXA1) is an anti-inflammatory protein reported to play a role in cell proliferation and apoptosis, and to be deregulated in breast cancer. The exact role of annexin A1 in the biology of breast cancer remains unclear. We hypothesized that the annexin A1 plays an oncogenic role in basal subtype of breast cancer by modulating key growth pathway(s).

**Methods:**

By mining the Cancer Genome Atlas (TCGA)-Breast Cancer dataset and manipulating annexin A1 levels in breast cancer cell lines, we studied the role of annexin A1 in breast cancer and underlying signaling pathways.

**Results:**

Our in-silico analysis of TCGA-breast cancer dataset demonstrated that annexin A1 mRNA expression is higher in basal subtype compared to luminal and HER2 subtypes. Within the basal subtype, patients show significantly poorer overall survival associated with higher expression of annexin A1. In both TCGA patient samples and cell lines, annexin A1 levels were significantly higher in basal-like breast cancer than luminal and Her2/neu-positive breast cancer. Stable annexin A1 knockdown in TNBC cell lines suppressed the mTOR-S6 pathway likely through activation of AMPK but had no impact on the MAPK, c-Met, and EGFR pathways. In a cell migration assay, annexin A1-depleted TNBC cells showed delayed migration as compared to wild-type cells, which could be responsible for poor patient prognosis in basal like breast cancers that are known to express higher annexin A1.

**Conclusions:**

Our data suggest that annexin A1 is prognostic only in patients with basal like breast cancer. This appears to be in part due to the role of annexin A1 in activating mTOR-pS6 pathway.

## Background

Breast cancer is a heterogeneous disease, and treatment options, response, and prognosis vary greatly with breast cancer subtype. Basal-like breast cancers represent about 10–15% of all breast cancers, and have a high potential for metastasis [1]. While basal- like breast cancer is defined by its genomic signature, the majority of these cancers are negative by immunohistochemistry for estrogen receptor (ER), progesterone receptor (PR), and Her2/neu, and hence are commonly referred to as triple negative breast cancer (TNBC). Currently no targeted therapies are available for this tumor subtype and thus these tumors are also characterized by a relatively lower survival rate.

Identification of biological markers of disease prognosis that can be targeted for therapy may help improve outcomes for basal like breast cancer patients. One such potential marker, annexin A1 (encoded by *ANXA1*), is a calcium-dependent phospholipid binding protein that shows phospholipase A2-inhibitory activities, is induced by glucocorticoids [2], and possesses anti-inflammatory activities [3]. To effect anti-inflammatory activities, annexin A1, which is expressed on the cytosolic face of the plasma membrane, is secreted by neutrophils upon cell adhesion to the endothelium, induces cell detachment, and inhibits transmigration of leukocytes [4]. ANXA1 expression has been reported to be deregulated in tumor development and progression, but the exact mechanisms remain unknown. To date, numerous reports have suggested differential expression of ANXA1 depending on cancer type: up-regulation in esophageal cancer [5], pancreatic cancer[6], skin squamous cell carcinoma [7], and colon cancer [8] and down-regulation in cervical cancer [9], oral squamous cell carcinoma [10], and prostate cancer [11]. Thus, annexin A1 seems to play an oncogenic role in some cancers and a tumor-suppressive role in others in a context specific manner.

There are conflicting reports in the literature about the role annexin A1 plays in breast cancer. Several studies have reported higher levels of annexin A1 expression in ductal carcinoma compared to normal ducts [12, 13]. Others have reported decreased annexin A1 expression in ductal carcinoma in situ and invasive ductal carcinoma compared to normal and benign tissues [14–17]. The role and regulation of annexin A1 in the biology and prognosis of breast cancer remain unclear and may be due to the lack of consideration of the different subtypes of breast cancer. Thus, in this study, we hypothesize that annexin A1 plays an oncogenic role in basal like breast cancer.

## Materials and Methods

### Ethics Statement

N/A

### TCGA breast cancer data mining

We evaluated the association between ANXA1 gene expression and overall patient survival in breast cancer dataset (TCGA, https://tcga-data.nci.nih.gov/tcga/) in a subtype-specific fashion. For each breast cancer subtype, Basal, Her2/neu-enriched, and luminal, specimens were first sorted according to the expression of the ANXA1 gene expression from the RNA Seq dataset (total patient samples = 890) of TCGA breast cancer, then association with overall survival was evaluated by comparing the top 50% of the specimens and the bottom 50% of the specimens using the log-rank test (p<0.05). Overall, survival significance was evaluated by employing the package survival [18] in the R statistical system.

### Cell culture

The following cell lines were obtained from the American Type Culture Collection (ATCC): i) TNBC lines MDA-MB-157, MDA-MB-436, MDA-MB-468, HCC70, BT-549, ii) ER+ /HER2/neu negative cell lines T-47D, ZR-75-1, MCF-7, MDA-MB-415, HCC1428, BT-483, iii) ER^+^/ Her2/neu^+^ cell lines BT474, and MDA-MB-361 and iv) ER-/Her2/neu^+^ cell lines AU565, HCC1954, and SKBR3. All cells except HS578T were cultured in DMEM-F12 containing 10% FBS. The TNBC line, HS578T also obtained from ATCC, was grown in DMEM with reduced NaHCO_3_ (ATCC) containing 0.1 mM insulin and 10% FBS. The ER^+^/ Her2/neu-overexpressing MCF7 (MCF7-Her18) cells were a kind gift from Dr. Elizabeth Mittendorf. All the cell lines used in here were strictly with in ten passages after buying from ATCC and thus were not authenticated again.

### Generation of stable annexin A1 knockdown cells

For annexin A1 knockdown, six lentiviral small hairpin RNA (shRNA) clones and a GIPZ non silencing lentiviral shRNA control clone (RHS 4348) were bought from Open Biosystems through The University of Texas MD Anderson Cancer Center’s shRNA and ORFeome Core Facility. The six annexin A1-silencing clones were as follows: clone A, V2LHS_112102, that generates a mature antisense transcript of sequence ATCTTCATCAGTTCCAAGG; clone B, V3LHS_413324, mature antisense sequence TCAGCTACATAGACATCTT; clone C, V3LHS_392260, mature antisense sequence AGCAGAGCTAAAACAACCT; clone D, V3LHS_413326, mature antisense sequence AGCTTGAGACCATCAAGGG; clone E, V3LHS_413325, mature antisense sequence AGAACAACTTGTATAGGGT; and clone F, V3LHS_392259, mature antisense sequence ATTTCTGAAACACTCTGCG. Stable MDA-MB-436 and MDA-MB-468 cell lines with annexin A1 knockdown were generated as described elsewhere [19]. Briefly, the A293T cell line was used to package the virus by transfecting the shRNA plasmids pCMV-Δr8.2 and pCMV-VSVG with Lipofectamine 2000 (Invitrogen) at a 10:10:2 ratio. The medium was replaced with fresh medium 18 hours later, and the supernatant containing the virus was collected 48 and 72 hours after the medium was changed. The supernatant was mixed with PEG-it precipitation solution (System Biosciences) and stored at 4°C for 24–48 hours. Virus was concentrated 100× by centrifuging the samples at 1500 × *g* for 30 minutes and resuspending the pellets in PBS. The virus-packaged annexin A1 shRNAs were then used to infect the cell lines. Positive clones were selected by using 2-μg/ml puromycin. After selection, short-term cultures of stable cell clones were maintained using 1 μg/ml puromycin.

### Transient transfections

For transient transfection studies, stable Annexin A1 shRNA clones D, E and F were transfected using Lipofectamine 2000 (Invitrogen Technologies) following the manufacturer's instructions. Briefly, Cells were plated on 6-well culture dishes and then cotransfected with 1.5 μg of the mTOR-containing plasmid (mTOR-pcDNA 3) or 1.5 μg of empty vector using 4 μl lipofectamine 2000. After a 6-h incubation in reduced serum medium optiMEM, the medium was replaced with DMEM-F12 supplemented with 10% FBS. Forty-eight hours after the transfection either cell lysates were prepared to extract proteins for westerns or the wells were processed for wound healing assay, as described later. pcDNA3-Flag mTOR wt was a gift from Dr. Jie Chen (Addgene plasmid # 26603) [20].

### Western blotting

Thirty to 40 μg of total cellular proteins was subjected to sodium dodecyl sulfate–polyacrylamide gel electrophoresis; transferred to Hybond ECL nitrocellulose (Amersham); and probed with either ANXA1, pAKT (S473), AKT, S6, or pS6 (S240/244), p44/42 MAPK, phospho- p42/44 MAPK (Thr202/Tyr204), mTOR, pmTOR (S2448), cmet, p-cmet (Tyr1234/1235), AMPK, Pampkα (Thr172), EGFR, pEGFR (Tyr1068) antibody or the loading control, vinculin. Proteins were detected by using the “Odyssey classical Imager” Infrared Imaging System (Li-Cor Biosciences). We were able to probe a single Western blot membrane for more than two proteins of interest (of different sizes, by cutting the membrane) and vinculin because of the ability of the Odyssey system to detect signals from antibodies raised in mouse and rabbit on the same membrane at separate wavelengths. As a result, in our Western blots, one common vinculin band is shown for multiple proteins if the proteins came from same membrane. Phospho proteins and total proteins were probed on different membranes because we found nitrocellulose membranes to be incompatible for re-probing followed by detection of fluorescence signal through odyssey in our hands. To ensure equal protein loading each membrane was probed with a loading control protein vinculin. Relative changes in phopho proteins levels were calculated by a two-step process: in the first step a ratio of each protein was calculated by normalizing against their respective vinculin. In the second step, a ratio of phospho protein with respect to the total protein was calculated to determine changes in phospho proteins.

### Scratch assay/wound-healing assay

MDA-MB-468 and MDA-MB-436 cells were plated in six-well dishes at a cell density of about 80% (500,000 cells/well) in DMEM-F12. Cells were scratched with a fresh sterile pipette tip (200 μl), and detached floating cells were washed out to ensure that they did not stick back to the wound. Subsequently, the cells were washed with PBS, and fresh cell culture medium was added to the wells. Pictures of the wound were taken at a 20× magnification, and the area of the wound was marked. Twenty-four hours later, pictures of the cells were taken in the same area. To quantify cell migration, the average widths of the wound were measured at time zero and at 24 hours (by using Image J) and the average distance covered by the untreated cells was set as 100% and the mean % distance covered by the annexin knock down cells clones and nonspecific shRNA clone was calculated with respect to the untreated cells. The following formula was used for this conversion: = (mean distance covered by the untreated cells/ mean distance covered by the annexin depleted clones (treated))*100.

### Electrophoretic mobility shift assay (EMSA) for NF-κB

To determine NF-κB activation, nuclear cellular proteins were analyzed by electrophoretic mobility gel shift assay (EMSA) as described elsewhere[21]. Briefly, 10 μg nuclear extracts were incubated with ^32^P-end-labeled 45-mer double-stranded NF-κB oligonucleotide (16 fmol of DNA) representing NF-**κ**B consensus sequence from the human immunodeficiency virus long terminal repeat, 5′-TTGTTACAAGGGACTTTCCGCTGGGGACTTTCCAGGGAGGCGTG G-3′ (NF-κB binding sites are underlined) for 15 min at 37°C, and the DNA-protein complex formed was separated from free oligonucleotide probe on 6.6% native polyacrylamide gels. An overnight exposure was obtained on radiographic film by placing it on dried gel in an autoradiography cassette.

### RNA extraction and QPCR

Total cellular RNA was extracted from exponentially growing cells by Trizol extraction (BioRad) method as described previously[22]. cDNA was prepared from RNA by using iscript cDNA synthesis kit (BioRad). The basal MMP9 mRNA levels were measured with respect to a loading control ribosomal protein L19 by SYBR green based QPCR method as described previously [22]. The primer sequences used for human MMP-9 were: forward, 5′-ttgacagcgacaagaagtgg-3′; and reverse, 5′-gccattcacgtcgtccttat-3′.

### Statistical analyses

Student’ unpaired t test was performed to measure the statistical difference between various groups. P values < 0.05 were considered statistically significant. Log Rank test was used to measure the statistical difference between the high annexin A1 and low annexin A1 groups for Kaplan-Meier curves. To measure the differences in expression of annexin A1 in breast cancer patients of different subtypes one-way ANOVA was used.

## Results

### High levels of annexin A1 mRNA are associated with poor prognosis in basal-like breast cancer

**Fig 1:**
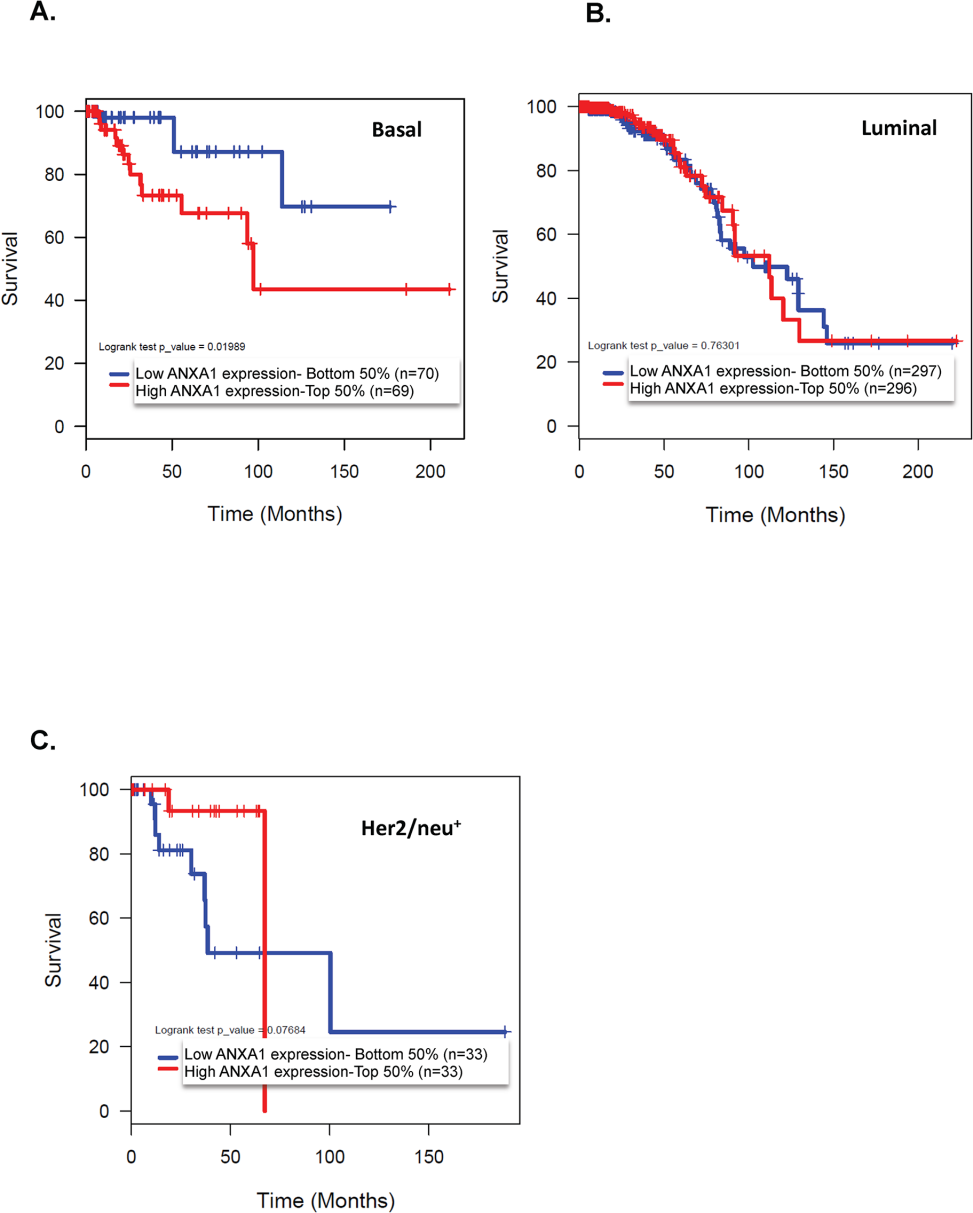
High levels of annexin A1 are associated with low overall survival rates in basal-like breast cancer. The Kaplan-Meier curves indicate the overall survival rates for basal-like (A), luminal (B), and Her2/neu^+^ (C) cancers. The p values refer to comparisons between patients with annexin A1 levels above the median (top 50%) and patients with annexin A1 levels below the median (bottom 50%); in the basal group, p = 0.019.

Our survival analysis of 890 TCGA breast cancer patient samples revealed that amongst the 139 patients with basal-like breast cancer tumors, high annexin A1 mRNA expression was associated with significantly shorter 5-year overall survival than patients with low annexin A1 expressing basal tumors (Fig 1A). Overall survival was 87% in low annexin A1 expressing patients with basal tumors at 100 months versus 43% in the high annexin A1 expressing group (p<0.001, Fig 1A). Whereas, in patients with both luminal and Her/neu positive tumors annexin A1 levels did not show any significant association with survival (Fig 1B & 1C). These data suggest a subtype specific role to annexin A1, and in particular an oncogenic role of annexin A1 in TNBC.

### Basal-like breast cancers express higher levels of annexin A1 protein than other subtypes

**Fig 2:**
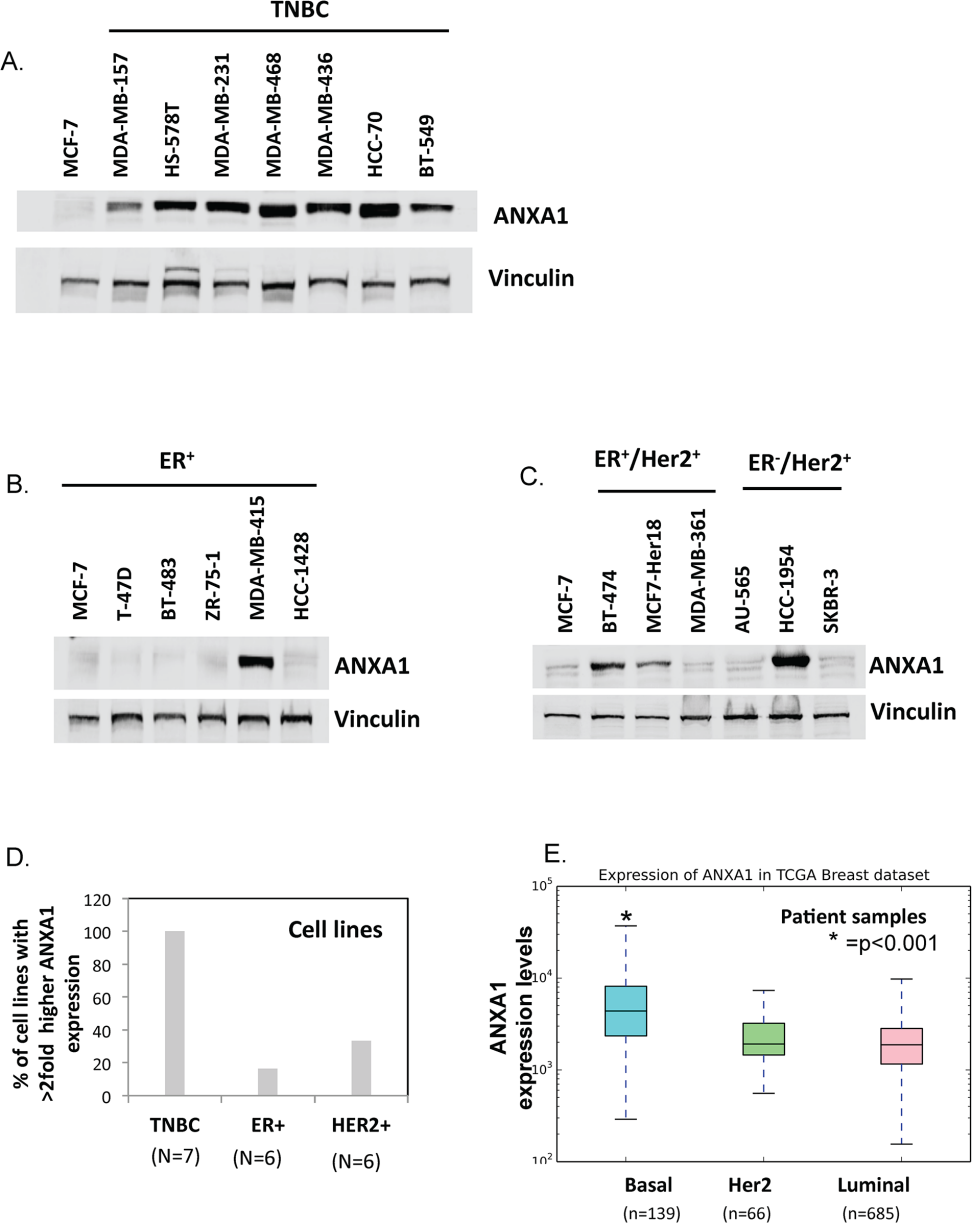
TNBC cell lines and basal-like breast cancer patients express high annexin A1. (A–C) Western blots showing annexin A1 protein expression relative to vinculin loading control in TNBC cell lines (A), ER^+^ cell lines (B), and ER-,Her2/neu^+^ and ER+, Her2/neu^+^ cell lines (C). (D) Proportion of cell lines in which annexin A1 protein was expressed at a level that was at least twice as high as the level in the MCF7 reference (ER^+^) cells. For this analysis, all the Her2+ lines (whether ER+/Her2+ or ER-/Her2+) were combined). (E) Box plots showing annexin A1 mRNA levels that are normalized by quantile normalization within basal, luminal, and HER2/neu+ breast cancers subtype patients. * indicates that basal subtype is statistically different than others at p<0.001. Cell line based experiments were repeated three times.

We looked at overall expression levels of annexin A1 protein using a panel of cell lines and annexin A1 mRNA using TCGA data. Our Western blot analysis of TNBC, ER^+^ (corresponding to luminal A like in TCGA dataset), and Her2/neu positive breast cancer cell lines revealed that TNBC cell lines had higher endogenous levels of annexin A1 protein than the non-TNBC cell lines. All seven TNBC cell lines tested expressed high annexin A1 levels (at least twice the level in the MCF7 reference cell line), whereas only one of five ER^+^ (20%), and two of six Her2/neu^+^ (33%) breast cancer cell lines had higher expression of annexin A1 relative to reference MCF7 cell line ((Fig 2A–2D, S1 Fig). Consistent with this laboratory based findings; annexin A1 mRNA levels were significantly higher in basal-like breast cancer patients (n = 139) than in samples from patients with luminal (n = 685) or Her2/neu^+^ positive (n = 66) cancers (TCGA breast cancer dataset) (Fig 2E). Fig 2E shows the annexin A1 levels that are normalized by quantile normalization within each breast cancer subtype.

### High annexin A1 expression activates breast cancer-relevant pathways in TNBC cell lines

**Fig 3:**
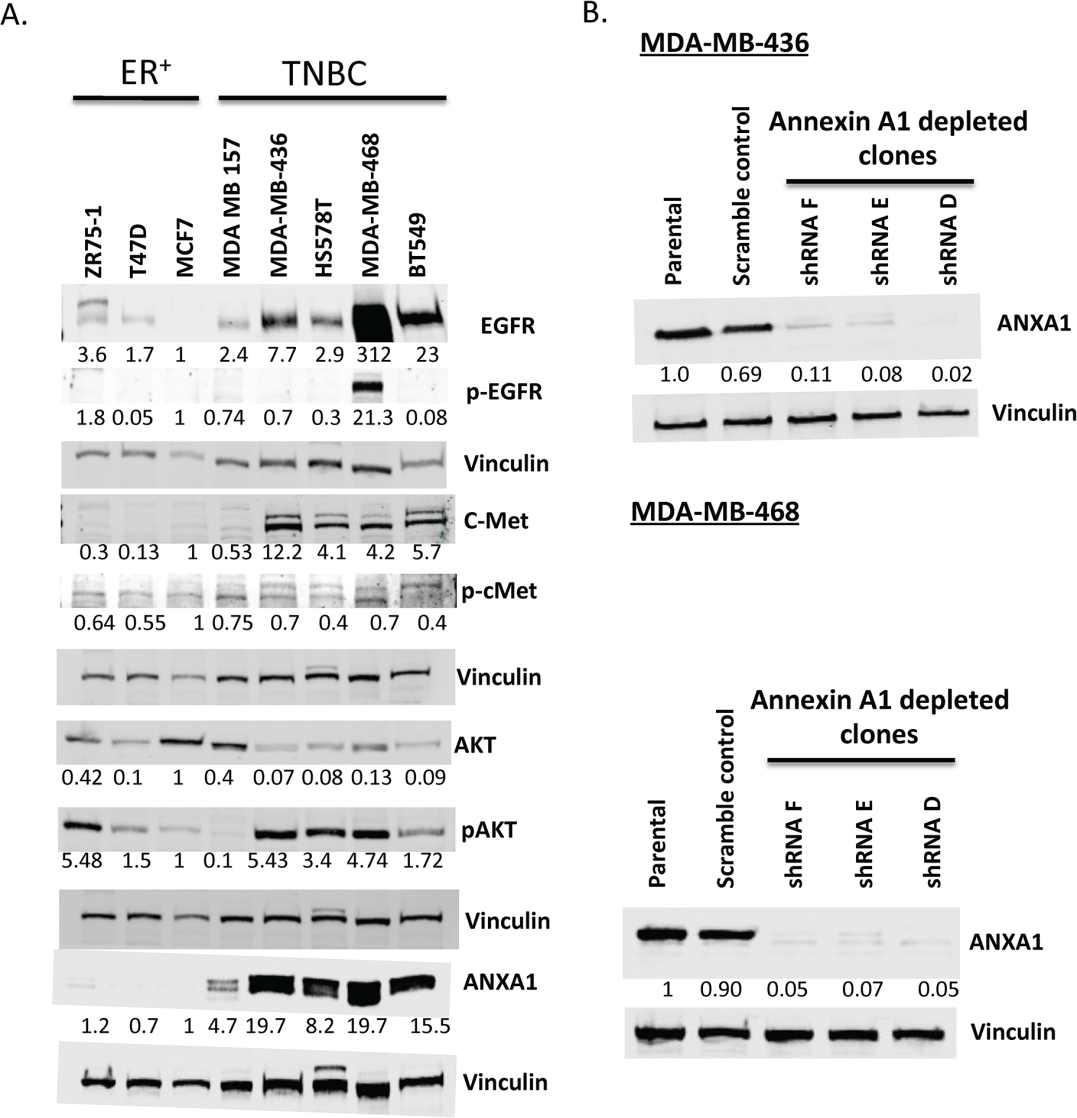
Activation status of oncogenic pathways and knockdown of annexin A1 in TNBC cell lines. (A) Western blot showing expression of EGFR, c-Met, pAKT, annexin A1, and MAPK relative to vinculin (loading control) in TNBC and ER^+^ cell lines. (B) Western blot showing annexin A1 knockdown in the MDA-MB-436 and MDA-MB-468 cell lines.—ve, scramble control. Representative figures from multiple experiments are shown here.

To investigate the mechanism by which high expression of annexin A1 contributes to poor TNBC prognosis, we studied endogenous expression of several breast cancer-relevant pathways, including the PI3K pathway, since TNBC have high levels of PI3K/AKT pathway activity resulting from a number of alterations including PI3KCA mutations and loss of PTEN and INPP4B [23]. We found TNBC cell lines expressing higher endogenous levels of annexin A1 to be associated with overactivation of EGFR, c-Met, and pAKT pathways compared to expression levels in ER^+^ breast cancer cell lines (Fig 3A).

### Annexin A1 knockdown inhibits mTOR-S6 signaling

**Fig 4:**
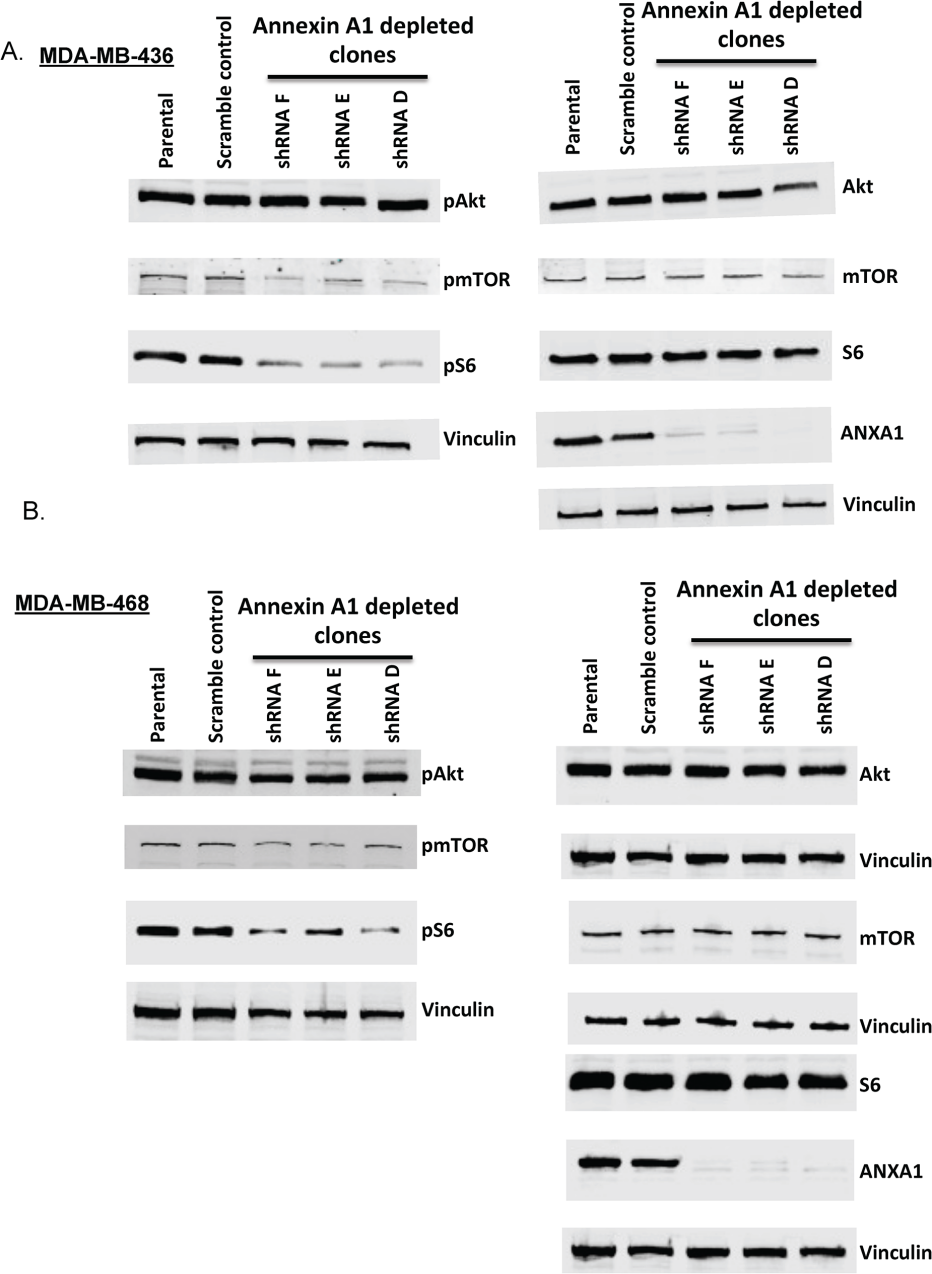
Annexin A1 knockdown blocks mTOR-S6 signaling. (A and B) Western blot showing levels of pAKT, AKT, pmTOR, mTOR, S6, pS6, and annexin A1 relative to vinculin (loading control) in MDA-MB-436 and MDA-MB-468 parental cells and annexin A1 silenced clones. Representative images from multiple experiments are shown. Quantitation of these westerns is summarized in S3 & S4 Figs

To determine whether the observed associations between high annexin A1and high EGFR, c-met, and pAKT levels in TNBC cells were causal, we generated annexin A1 knock down in the TNBC lines, MDA-MB-436, and MDA-MB-468. Six different clones with stable knockdown of annexin A1 in MDA-MB-436 cell background were generated initially, along with a scramble control shRNA-containing cell line under control of a constitutive cytomegalovirus promoter. Of the six clones tested, three (clones D, E, and F) showed near complete knockdown of annexin A1, as confirmed by Western blotting and fluorescence imaging in live cell cultures (the vector used to create the shRNA clones is tagged with green fluorescent protein) (Fig 3B and S2 Fig). Analyses of these clones revealed that stable annexin A1 knockdown inhibited the mTOR-S6 pathway (Fig 4) in MDA-MB-436 and MDA-MB-468 cells. Specifically, the average inhibition in phosphorylation of mTOR across three annexin A1 depleted clones was 35% in MDA-MB-436 cells and 37% in MDA-MB-468 cells relative to scramble shRNA control (p<0.01, S3 and S4 Figs). Similarly a significant inhibition in phosphorylation of ribosomal protein S6 was noted across three annexin A1 knock down clones (53% in MDA-MB-436 and 37% in MDA-MB-468) (p<0.01, Fig 4 and S3 & S4 Figs). Although annexin A1 depletion led to a significant inhibition in pEGFR levels in MDA-MB-468 cell clones (p<0.01, S4 and S5 Figs) relative to scramble shRNA control, this inhibition could not be tested in MDA-MB-436 cells because of undetectable levels of pEGFR at baseline (data not shown). In addition, annexin A1 knock-down did not inhibit total levels or phosphorylation of AKT, MAPK, or c-Met (Fig 4 and S3, S4 and S5 Figs) as compared to the scramble shRNA control. Next, we asked if the inhibition in mTOR-S6 signaling caused by annexin A1 knock down is mediated by NF-κB, a pathway that is known to cross talk with mTOR [24]. By performing electrophoretic mobility shift assay, we did not find any change in NF-κB activation in nuclear lysates of wild type and annexin A1 depleted cell clones in either MDA-MB-436, or MDA-MB-468 cells (S6 Fig) indicating that suppression in mTOR-S6 signaling in annexin A1 depleted cells is not mediated by NF-κB and other mechanisms may be at play.

### mTOR pathway suppression by annexin A1 depletion is associated with the activation of AMPK

**Fig 5:**
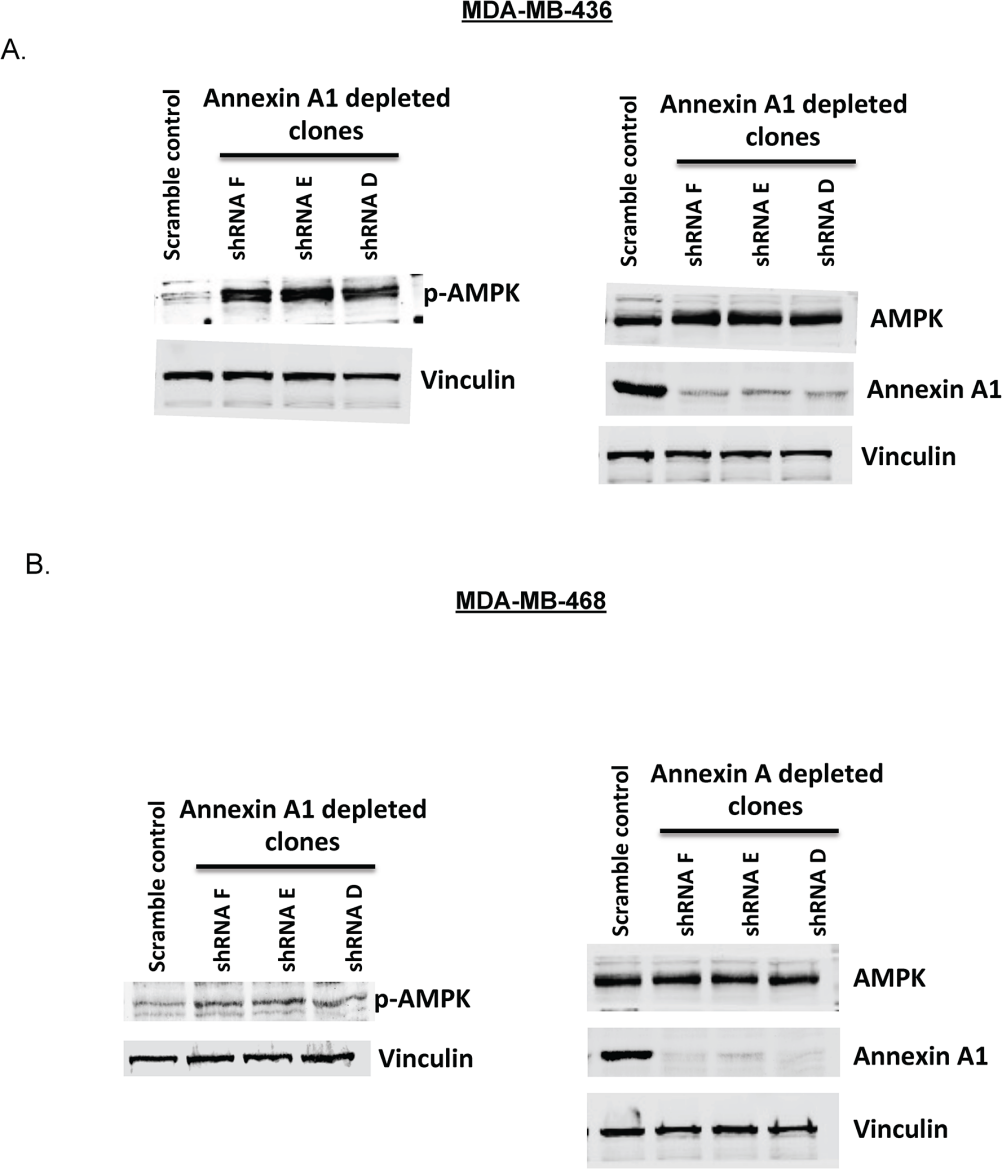
Annexin A1 knockdown activates AMPK. (A and B) Western blot showing levels of pAMPK, AMPK and annexin A1 relative to vinculin (loading control) in MDA-MB-436 and MDA-MB-468 parental cells and annexin A1 silenced clones. Representative images from multiple experiments are shown. Quantitation of these westerns is summarized in S3 & S4 Figs

Annexin A1 has been reported to be activated under stress conditions [25], and so is AMP- activated protein kinase (AMPK). The latter is also known to suppress mTORC1 (a multi-protein complex consisting of mTOR, raptor and mLST8) activity under stress conditions such as hypoxia or DNA damage and has a role in down regulating protein synthesis and arresting growth [26]. Thus we tested the possibility that annexin A1 works through a stress pathway, activating AMPK to suppress downstream pathways such as mTOR. Indeed, the annexin A1 depleted cell clones showed an increase in phosphorylation of AMPK (Fig 5). Specifically, there was at least 1.8 fold increase (p<0.01) in phosphorylation of AMPKα across all three annexin A1 clones relative to scramble shRNA control in both MDA-MB-436 and MDA-MB-468 (S3 and S4 Figs) cells.

### Annexin A1 knockdown inhibits breast cancer cell migration

**Fig 6:**
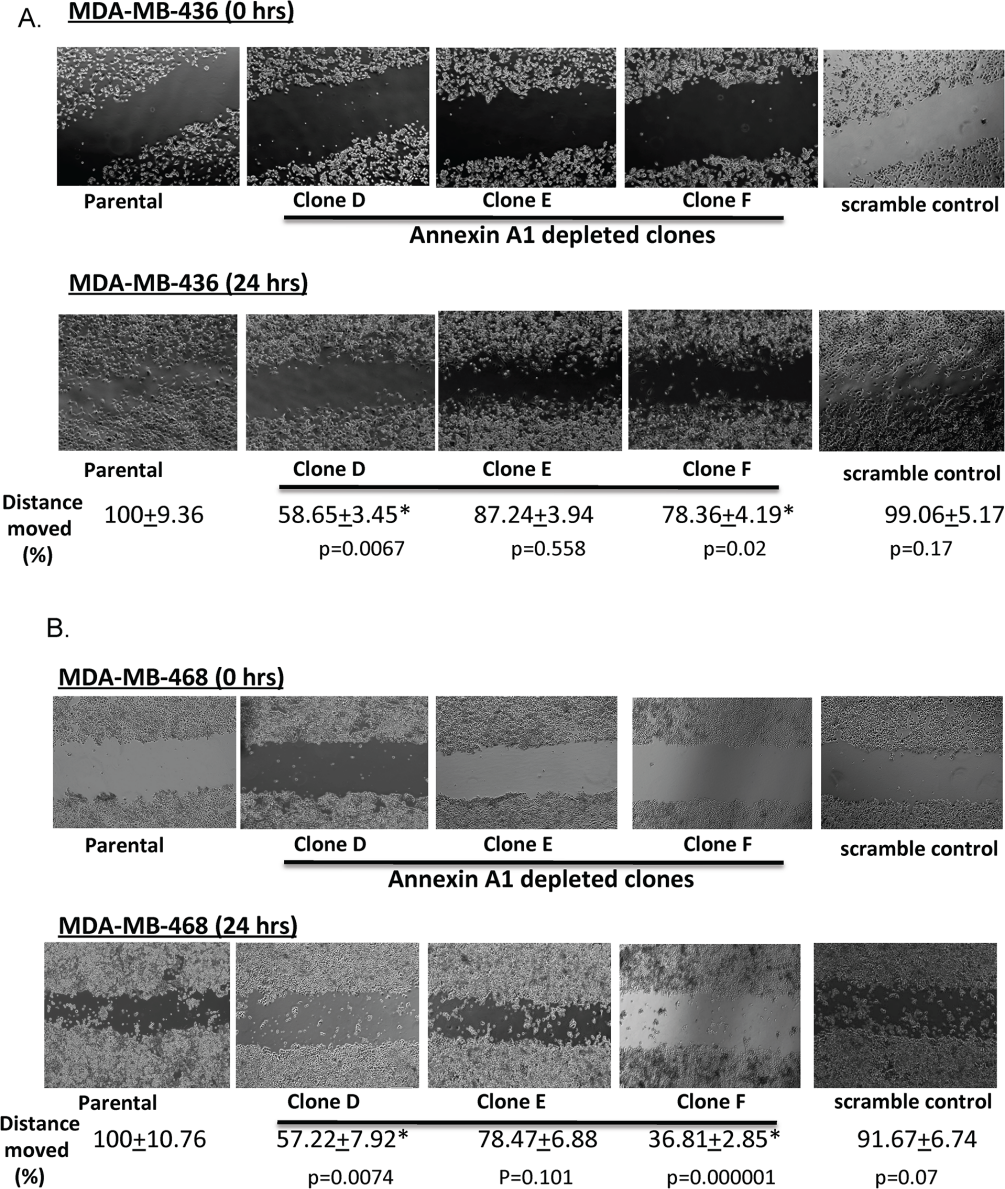
Annexin A1 depletion inhibits cell migration in TNBC cells. Migration was measured by scratch assays of annexin A1 shRNA-transfected MDA-MB-436 (A) and MDA-MB-468 (B) cells. The mean distances covered in 24hrs (in %, relative to the untreated cells, with standard errors) are shown. Scramble clone indicates non-silencing shRNA. Experiments were repeated three times.

**Fig 7:**
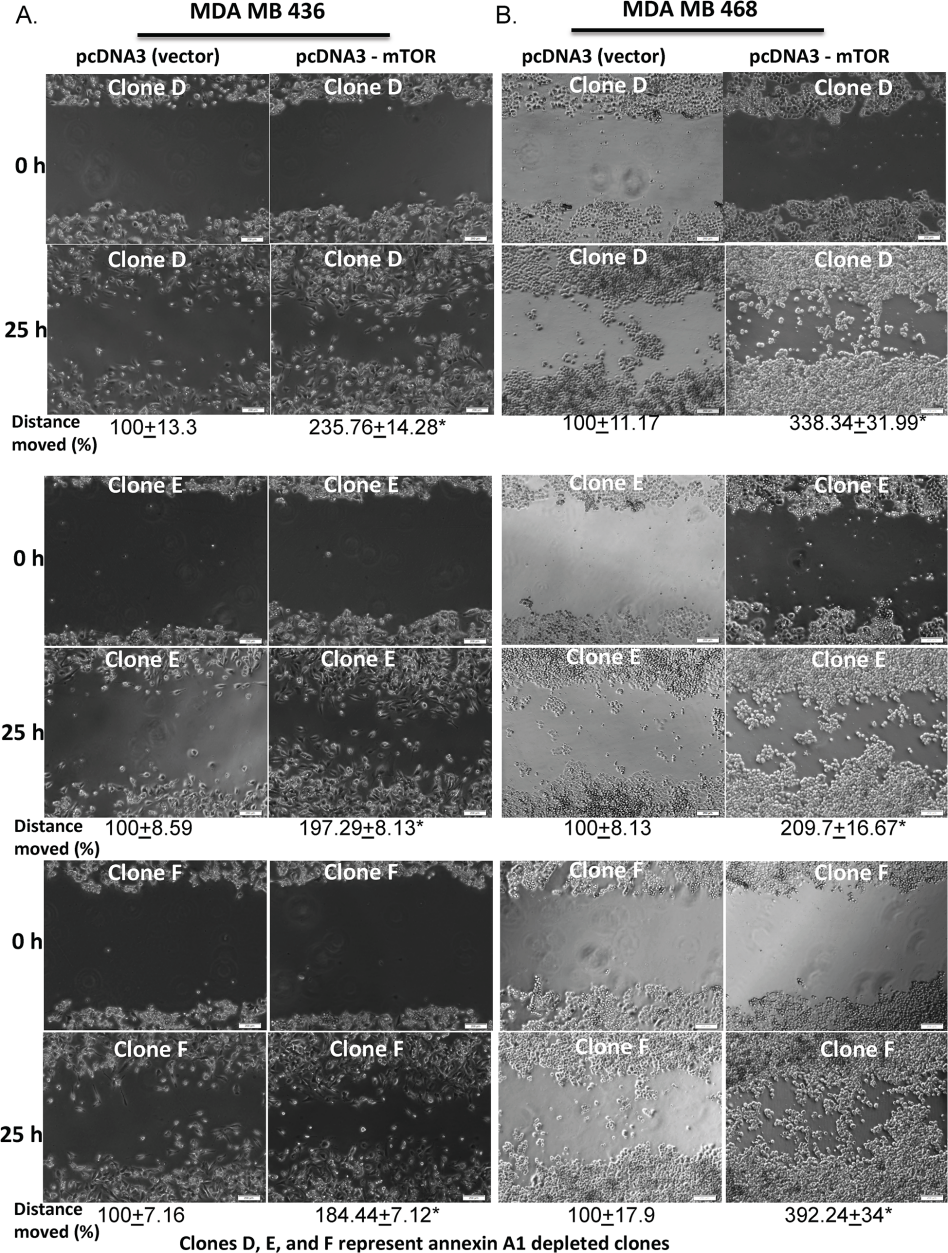
Ectopic expression of mTOR reverses impaired cell migration in annexin A1 deficient TNBC cells. Migration was measured by scratch assays of annexin A1 shRNA-transfected with vector control plasmid or with a pcDNA3-mTOR plasmid in MDA-MB-436 (A) and MDA-MB-468 (B) cell background. The mean distances covered in 25hrs (in %, as compared to the vector control treated cells, with standard errors) by the cells are shown. * indicates p<0.001 of mTOR transfected cell clones in comparison to vector control cells. Experiments were repeated three times.

To determine whether the inhibition of mTOR-S6 signaling caused by annexin A1 knockdown has any functional relevance, we investigated the effects of annexin A1 knockdown on cancer cell migration. A scratch assay showed that migration was impaired in the annexin A1 knockdown with 2 of the 3 MDA-MB-468 and MDA-MB-436 cell clones having overall migration distances that were significantly shorter (on average 21% to 63%) than in wild-type cells (p<0.05; Fig 6). One of the annexin silenced clone (clone E) in both cell lines, did not show a statistically significant impairment in cell migration but still had a trend towards impaired cell migration. To further demonstrate that impairment in cell migration exhibited by annexin A1 depleted cells is directly mediated through mTOR, we performed a gain of function experiment by overexpressing flag tagged full-length mTOR gene (in pcDNA 3 vector backbone) in annexin A1 depleted cells. mTOR transfected cells showed an increased mTOR protein expression (S7 Fig) and significantly increased cell migration as compared to vector controls (about 1.8- to 3.9-fold compared to controls, p< 0.001) (Fig 7A & 7B). These results indicate that suppression in mTOR signaling caused by annexin A1 depletion directly impairs cell migration in TNBC cells.

## Discussion

In this study, we sought to clarify the role of annexin A1 in the biology of breast cancer by examining its role in basal like subtype. We found that basal like tumors express the highest levels of annexin A1 and that this high expression of annexin A1 is correlated with poor prognosis. Using cell line models of annexin A1 depletion, we demonstrated for the first time that annexin A1 is associated with activation of mTOR-S6 signaling likely to be mediated through AMPK thus providing a mechanism by which high annexin A1 expression may result in the poorer survival outcomes of women with basal like breast cancer.

In contrast to our findings, Wang et al reported that annexin A1 appeared to be functioning as a tumor suppressor with low expression levels corresponding with poorer survival [27]. In a series of 135 cancer cases, they also found no specific association between ER, PR and Her2/neu expression and annexin A1 levels. Yom et al reported that although loss of annexin A1 was seen across the continuum of progression from DCIS to invasive cancer; amongst invasive cancer cases those with high expression of annexin A1 had poorer prognosis [17]. Although this was not born out on multivariate analysis, data from Yom et al would suggest that annexin A1 functions as an oncogene [17]. In both these series, the number of basal like tumors was limited and thus a robust analysis of annexin A1 and its association with subtype was not feasible. Our findings that ER+ tumors are generally low in expression of annexin A1 and that this gene is highly expressed in the basal subtype may explain the findings from prior reports. Because ER+ tumors account for the majority of all breast cancer cases, a review of breast cancer without consideration to subtype would support the view that annexin A1 levels in general are low in breast cancer, suggesting a role as a tumor suppressor. However, when viewed through the lens of tumor subtype, the full relevance and impact of annexin A1 becomes clear. Our data suggests that annexin A1 functions as an oncogene in TNBC subtype. In the other subtypes of breast cancer annexin A1 did not seem to play a role, as it did not predict prognosis in patients with ER+ and Her2/neu positive tumors in TCGA breast cancer samples. These findings are in concordance with those reported by de Graauw *et al* [28], who showed that taking into account annexin A1 levels can help physicians to accurately diagnose basal-like breast cancers in patients, the authors also found annexin A1 to promote metastasis in mice. Building on the link between annexin A1 and basal-like breast cancers that was described by de Graauw *et al*, we have demonstrated that high expression of annexin A1 has functional implications and is associated with shorter survival in basal-like breast cancer patients.

Our findings also provide the first evidence for the role of annexin A1 in regulation of mTOR-S6 signaling. The mTOR pathway and its downstream effectors ribosomal protein S6 kinase (S6K), and S6 are known to play a role in development and cancer by regulating translation of mRNA transcripts containing oligopyrimidine tract in their 5’ untranslated regions [29–31]. Phosphorylated S6 (pS6) is also reported to be over activated in several cancers and associated with cancer progression [32]. In addition, pS6 has been reported to be a marker of the mTOR activity, and predictive of early clinical response to targeted mTOR therapy [33, 34]. Although, mTOR has been previously reported to mediate growth factor induced cell migration in a wide variety of cell lines [35–39], our study has further added that mTOR signaling is regulated by annexin A1 leading to alterations in cell migration in TNBC cells. Previously, mTORC1, a complex containing mTOR itself, has been reported to regulate cell migration via S6K1 or 4E-BP1 pathways [36]. S6K pathway in turn is reported to cause alterations in F-actin reorganization, focal adhesion formation, tissue remodeling through the proteolytical digestion of extracellular matrix via up-regulation of MMP-9 [39–41]. In contrast, the effects of 4E-BP1 on cell migration are reported to be mediated through changes in mRNA translation and protein synthesis of a wide array of targets, including cyclinD1 and MMP-9 [42]. Thus, S6K1 and 4E-BP1 (two downstream effectors of mTOR pathway) seem to regulate multiple processes and targets including regulating the expression and activity of MMP-9 during migration. Using MDA-MB-231, a TNBC cell line, Kang et al [43] have previously shown annexin A1 to regulate MMP9 expression at both RNA and protein level. In our efforts to determine the involvement of MMP9 in cell migration in the present study, we observed a decrease in MMP9 mRNA levels in annexin A1 depleted clones (S8 Fig), but the alterations in MMP9 protein levels and activity could not be confirmed by immunofluorescence or zymography as the endogenous levels of MMP9 in MDA-MB-436 and MDA-MB-468 cells were barely detectable and seem to be below the detection limit of the western blotting and zymography (Data not shown). Therefore, we speculate that the impairment in cell migration caused by decreased mTOR-S6 signaling in our annexin A1 depleted TNBC cells could be mediated through other mechanisms as detailed above. In concordance with our studies, previously, Bist *et al* [44] have also reported ectopic expression of annexin A1 to increase cell migratory properties of TNBC cells albeit through a NF-κB dependent mechanism that we did not find to be implicated in our study (S6 Fig). Very elegantly, Bist *et al* further demonstrated annexin A1 deficiency to reduce metastasis and improve survival in mice models, which is in support of our findings that higher annexin A1 correlates with poor overall survival in basal like breast cancer patients [44].

Annexin A1, a protein that is mainly expressed on the cytosolic side of the plasma membrane, endosomal and phagosomal membranes[45–47], is likely to regulate mTOR (that is known to be present in both cytoplasmic or nuclear locations as a part of mTORC1 complex, [48]) through AMPK. Annexin A1 has been known as a stress sensor and upon oxidative stress has been reported to translocate to the nucleus[25]. AMPK is also known as a stress induced protein and is known to feed into the mTOR pathway [49, 50]. Indeed we found AMPK to be activated through phosphorylation in our annexin depleted TNBC cells. If annexin A1 directly interacts with AMPK to regulate mTOR signaling or it does indirectly is still unknown, one possibility is through activation of pATM that is known to be activated upon stress and is also known to activate AMPK[51]. And since annexin A1 is also known to play anti-inflammatory roles[52], its depletion in TNBC cells could lead to more stress that could trigger the activation of pATM, pAMPK and mTOR suppression. High annexin A1, low stress, and high mTOR-S6 signaling might seem contradictory in TNBC but there are several molecules that play distinctive roles in different stages of development and cancer and it is the overall effect of these alterations that determines the outcome of the process. This annexin-AMPK-mTOR-cell migration axis that we have described in here potentially explains the association between high annexin A1 levels and poor breast cancer prognosis in basal like breast cancer.

## Conclusions

Our data suggest that basal like tumors express high levels of annexin A1, which is associated with poor prognosis. However, the role of annexin A1 in the other subtypes of breast cancer is not as clear. Our Annexin A1 depletion in TNBC cell lines further suggests its tumorigenic role by promoting tumor cell migration through increased mTOR signaling.

## Supporting Information

S1 Fig Normalization with 2 different loading control proteins (vinculin and β-actin) show TNBC cell lines to express high annexin A1. (A–C) Western blots showing annexin A1 expression levels relative to vinculin or β-actin (loading control) in TNBC cell lines (A), ER^+^ cell lines (B), and ER-,Her2/neu^+^ and ER+, Her2/neu^+^ cell lines (C).
(TIFF)

S2 Fig Knockdown of annexin A1 in TNBC cell lines. Immuno fluorescence detection of annexin A1 shRNAs (clones D, E, and F, tagged with green fluorescent protein) and scramble control shRNA in MDA-MB-436 cells.
(TIFF)

S3 Fig Effect of annexin A1 knockdown on oncogenic pathways in MDA-MB-436. (A, B, C) Bar graphs showing the ratios of indicated phopsho protein to the total protein. Each protein was normalized first with its respective vinculin loading control and then to its scramble (negative) control [(protein of interest in annexin A1 clone/vinculin)/(protein of interest in negative clone/vinculin)]. A ratio was then taken of the normalized phoshoprotein to the normalized total protein. The bar length represents average values from 3 individual annexin A1 clones and 2 separate membranes. The error bars show SEM, and * indicates p<0.01.
(TIFF)

S4 Fig Effect of annexin A1 knockdown on oncogenic pathways in MDA-MB-468. (A, B, C) Bar graphs showing the ratios of indicated phopsho protein to the total protein. Each protein was normalized first with its respective vinculin loading control and then to its scramble (negative) control [(protein of interest in annexin A1 clone/vinculin)/(protein of interest in negative clone/vinculin)]. A ratio was then taken of the normalized phoshoprotein to the normalized total protein. The bar length represents average values from 3 individual annexin A1 clones and 2 separate membranes. The error bars show SEM, and * indicates p<0.01.
(TIFF)

S5 Fig Effect of annexin A1 knockdown on oncogenic pathways. (A and B) Western blot showing levels of pEGFR, pMAPK, p c-met, EGFR, MAPK, and c-met relative to vinculin (loading control) in MDA-MB-436 and MDA-MB-468 parental cells and annexin A1 silenced clones. Representative images from multiple experiments are shown.
(TIFF)

S6 Fig Annexin A1 depletion does not affect constitutively active NF-κB in TNBC cells. NF-κB binding was measured by performing EMSA of wild type and annexin A1 shRNA-transfected MDA-MB-436 and MDA-MB-468 nuclear proteins. The NF-κB binding, non-specific binding and free probes are shown. Scramble clone indicates non-silencing shRNA. The results shown are representative of 3 independent experiments.
(TIFF)

S7 Fig mTOR overexpression in annexin A1 depleted cells. Western blot showing overexpression of flag tagged mTOR, and loading control vinculin in clone D, E, and F of MDA-MB-436 cells.
(TIFF)

S8 Fig Annexin A1 knock down inhibits MMP9 expression. mRNA levels of MMP9 measured by QPCR in wild type and annexin A1 depleted clones of MDA-MB436 and MDA-MB-468 cells. All annexin A1 depleted clones were significantly different (p<0.05) relative to untreated/ parental cells. Experiments were repeated three times.
(TIFF)
