# Supplementary figures and images for: Annexin A1 Preferentially Predicts Poor Prognosis of Basal-Like Breast Cancer Patients by Activating mTOR-S6 Signaling
Source: PLoS One. 2015 May 22;10(5):e0127678. doi: 10.1371/journal.pone.0127678 (PMC4441370; doi:10.1371/journal.pone.0127678)

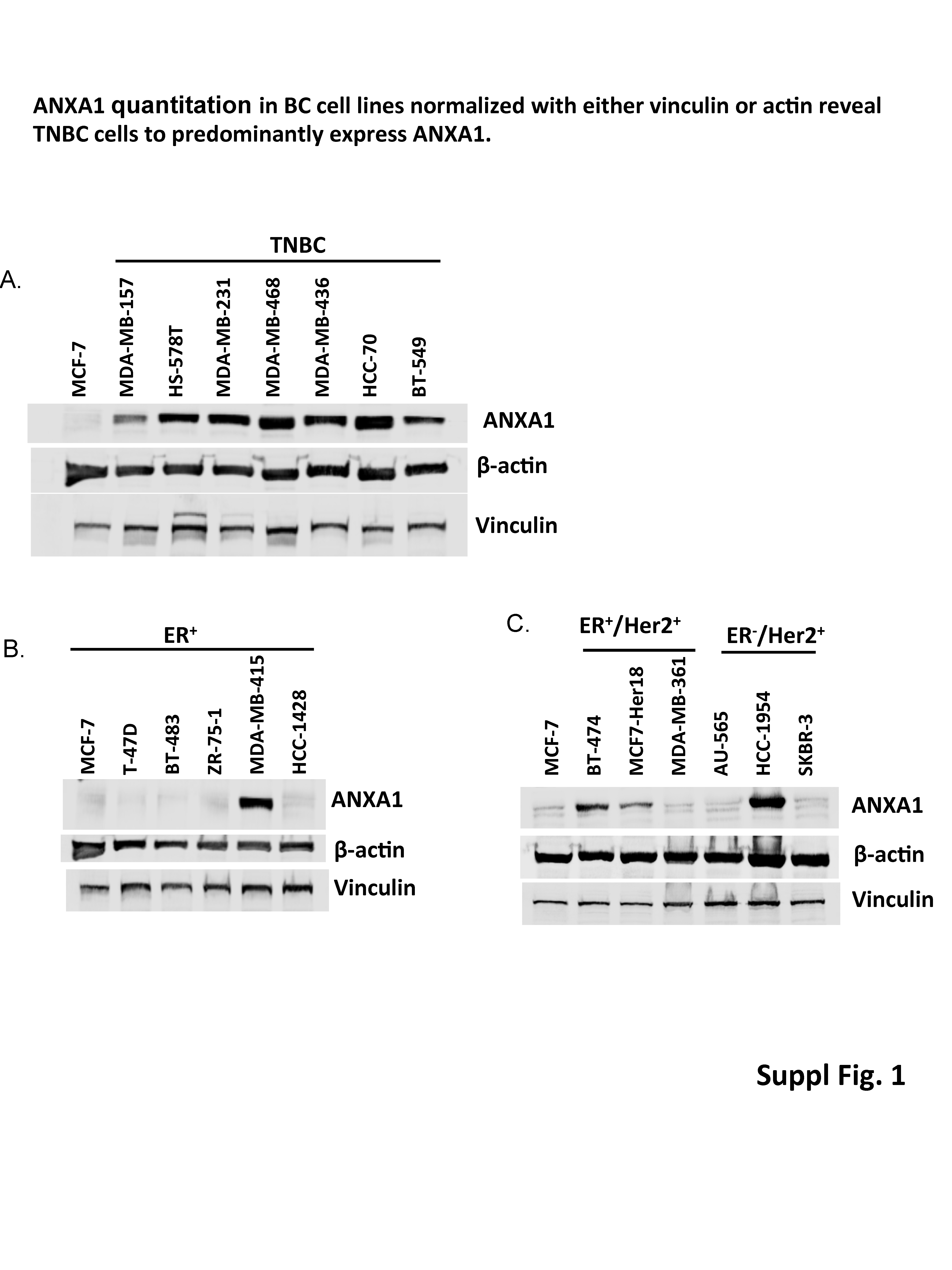

Supplement: S1 Fig — (A–C) Western blots showing annexin A1 expression levels relative to vinculin or β-actin (loading control) in TNBC cell lines (A), ER+ cell lines (B), and ER-,Her2/neu+ and ER+, Her2/neu+ cell lines (C). (TIFF) [file pone.0127678.s001.tiff]

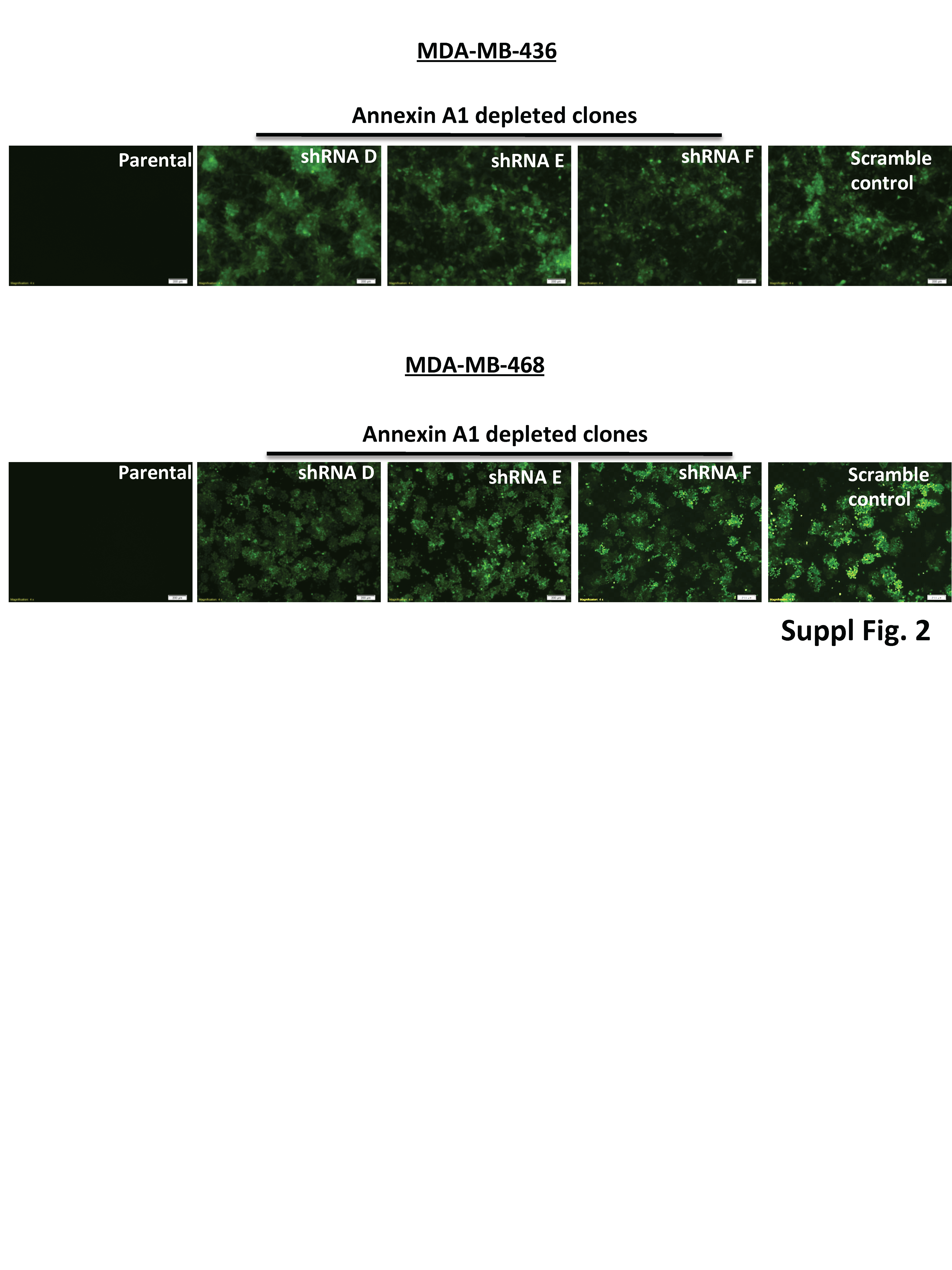

Supplement: S2 Fig — Immuno fluorescence detection of annexin A1 shRNAs (clones D, E, and F, tagged with green fluorescent protein) and scramble control shRNA in MDA-MB-436 cells. (TIFF) [file pone.0127678.s002.tiff]

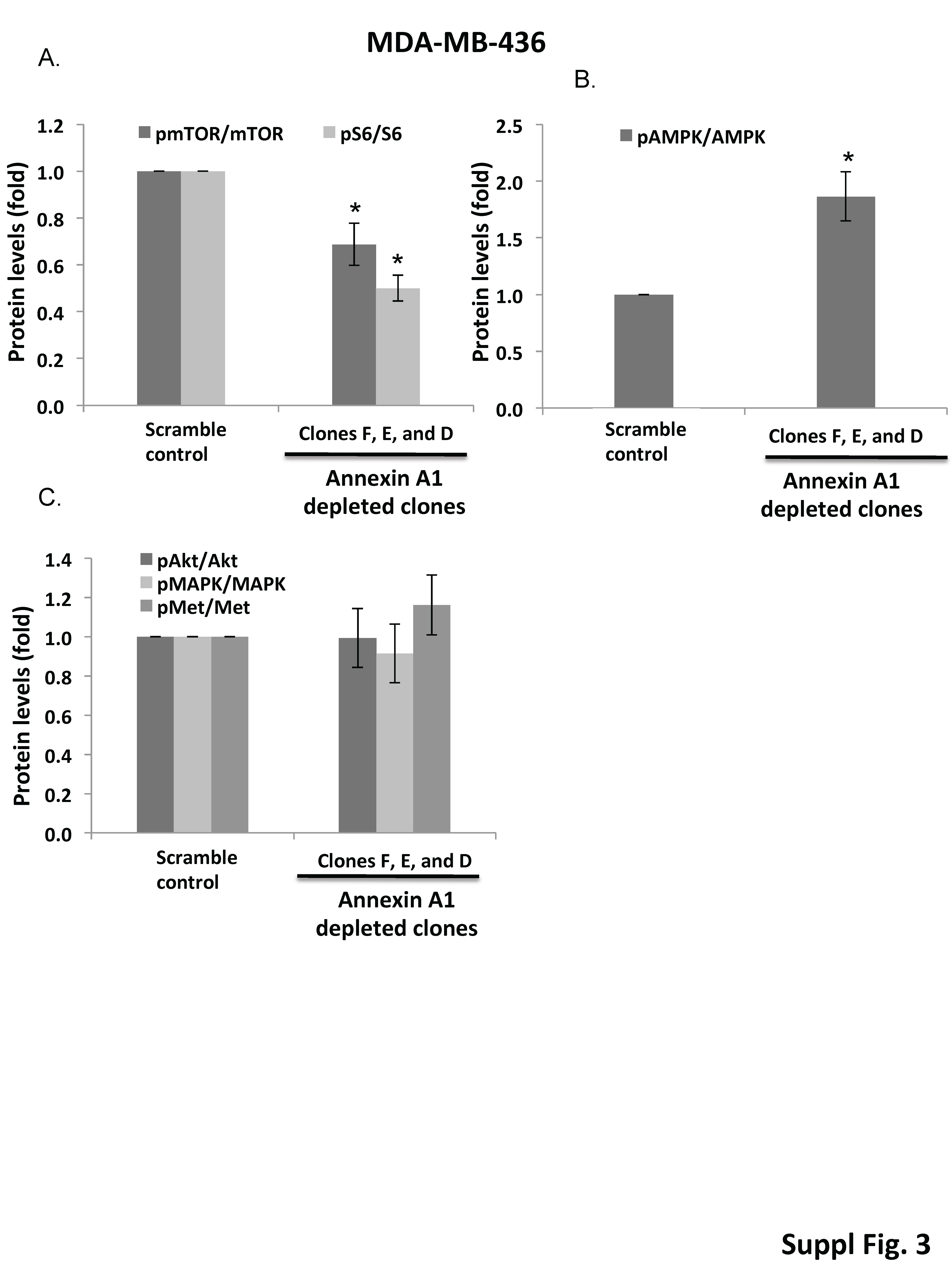

Supplement: S3 Fig — (A, B, C) Bar graphs showing the ratios of indicated phopsho protein to the total protein. Each protein was normalized first with its respective vinculin loading control and then to its scramble (negative) control [(protein of interest in annexin A1 clone/vinculin)/(protein of interest in negative clone/vinculin)]. A ratio was then taken of the normalized phoshoprotein to the normalized total protein. The bar length represents average values from 3 individual annexin A1 clones and 2 separate membranes. The error bars show SEM, and * indicates p<0.01. (TIFF) [file pone.0127678.s003.tiff]

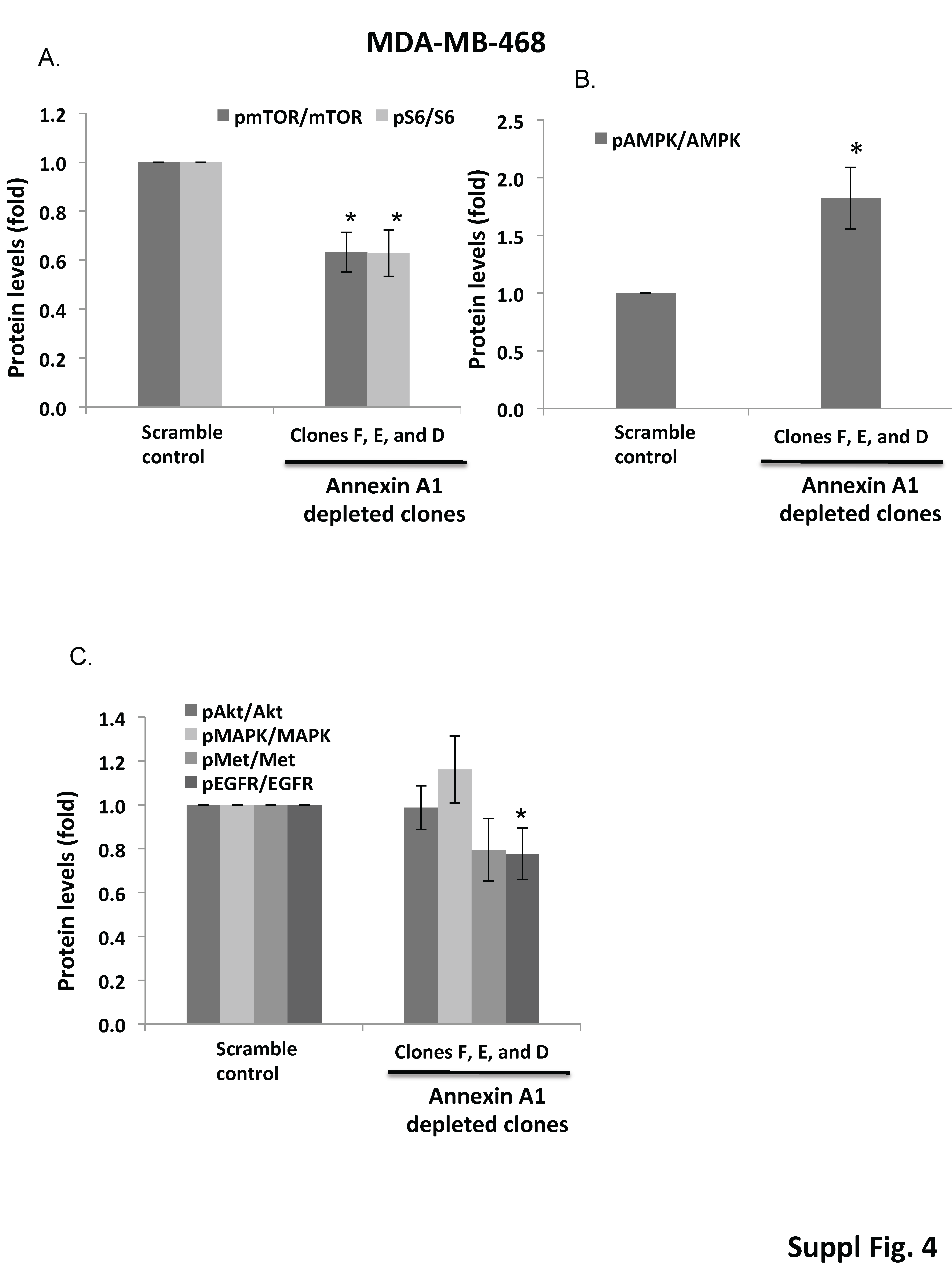

Supplement: S4 Fig — (A, B, C) Bar graphs showing the ratios of indicated phopsho protein to the total protein. Each protein was normalized first with its respective vinculin loading control and then to its scramble (negative) control [(protein of interest in annexin A1 clone/vinculin)/(protein of interest in negative clone/vinculin)]. A ratio was then taken of the normalized phoshoprotein to the normalized total protein. The bar length represents average values from 3 individual annexin A1 clones and 2 separate membranes. The error bars show SEM, and * indicates p<0.01. (TIFF) [file pone.0127678.s004.tiff]

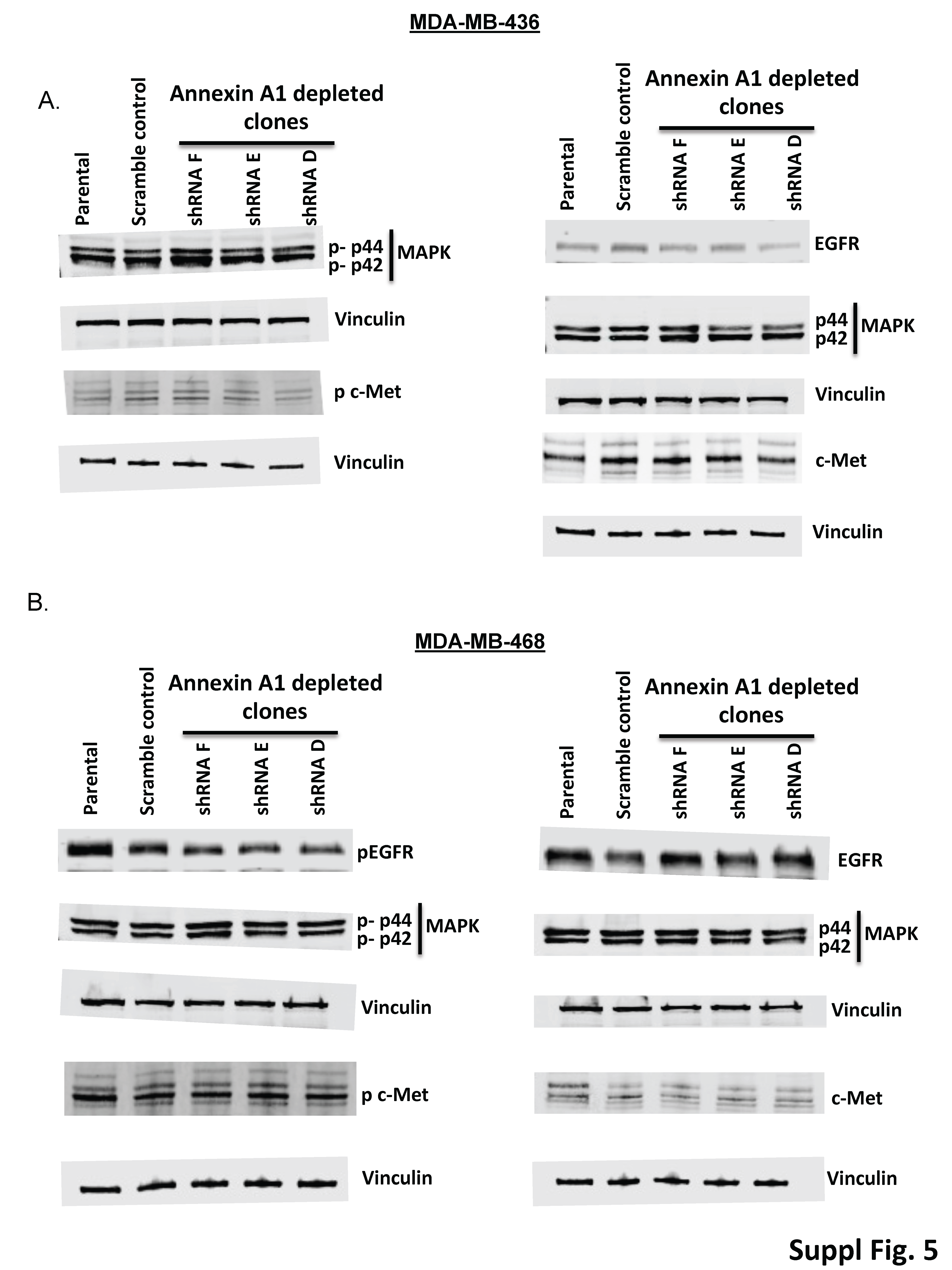

Supplement: S5 Fig — (A and B) Western blot showing levels of pEGFR, pMAPK, p c-met, EGFR, MAPK, and c-met relative to vinculin (loading control) in MDA-MB-436 and MDA-MB-468 parental cells and annexin A1 silenced clones. Representative images from multiple experiments are shown. (TIFF) [file pone.0127678.s005.tiff]

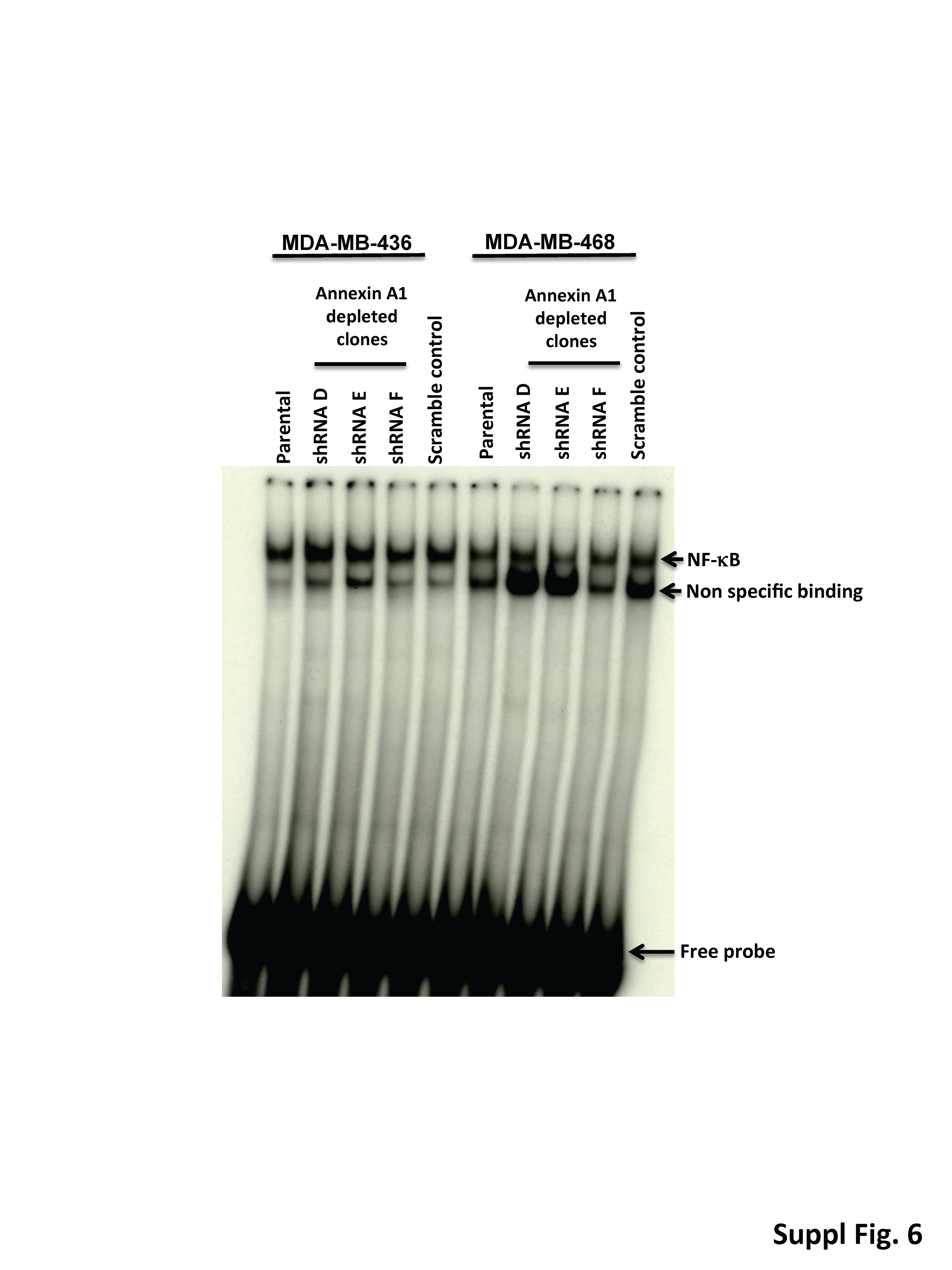

Supplement: S6 Fig — NF-κB binding was measured by performing EMSA of wild type and annexin A1 shRNA-transfected MDA-MB-436 and MDA-MB-468 nuclear proteins. The NF-κB binding, non-specific binding and free probes are shown. Scramble clone indicates non-silencing shRNA. The results shown are representative of 3 independent experiments. (TIFF) [file pone.0127678.s006.tiff]

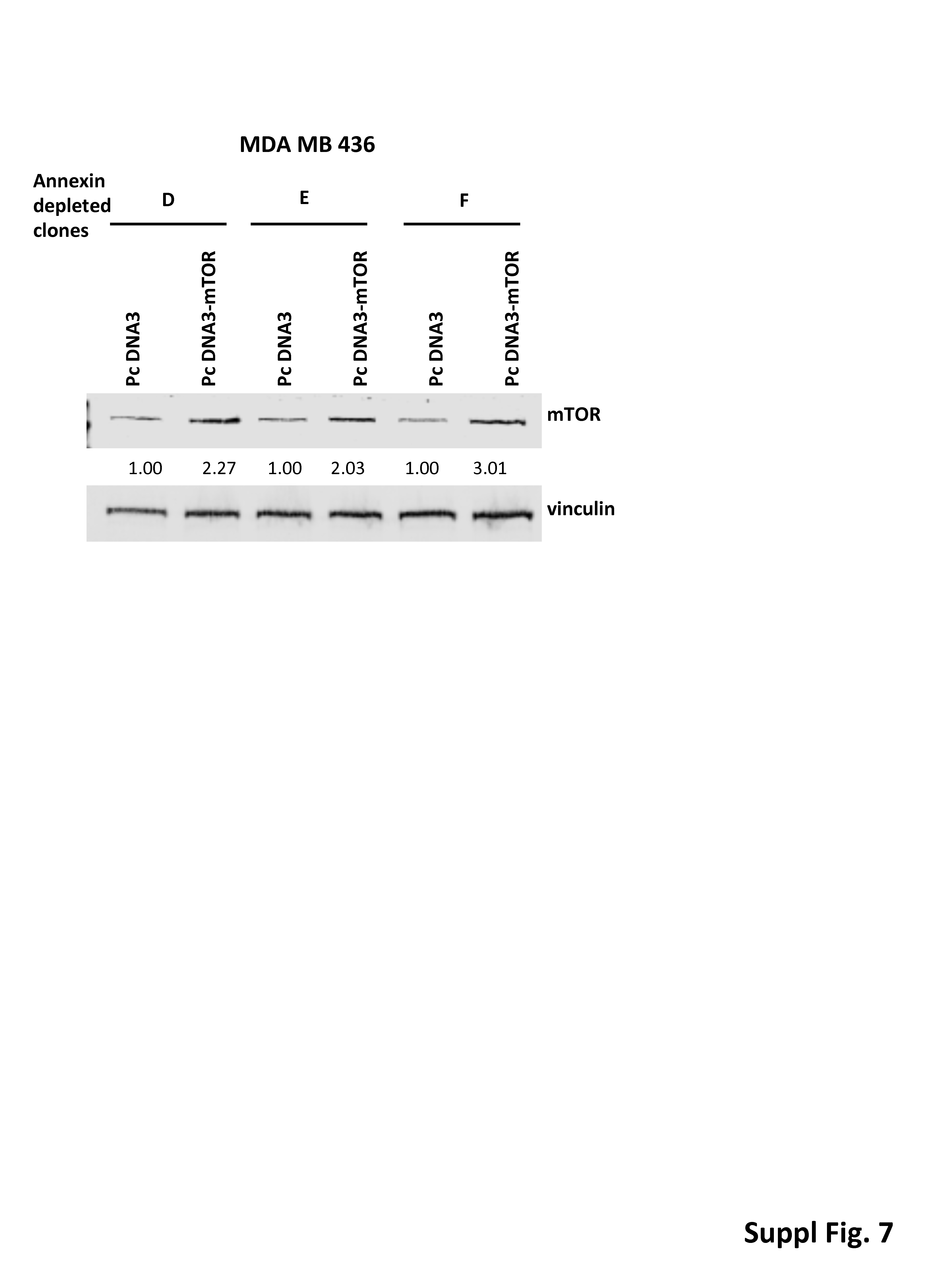

Supplement: S7 Fig — Western blot showing overexpression of flag tagged mTOR, and loading control vinculin in clone D, E, and F of MDA-MB-436 cells. (TIFF) [file pone.0127678.s007.tiff]

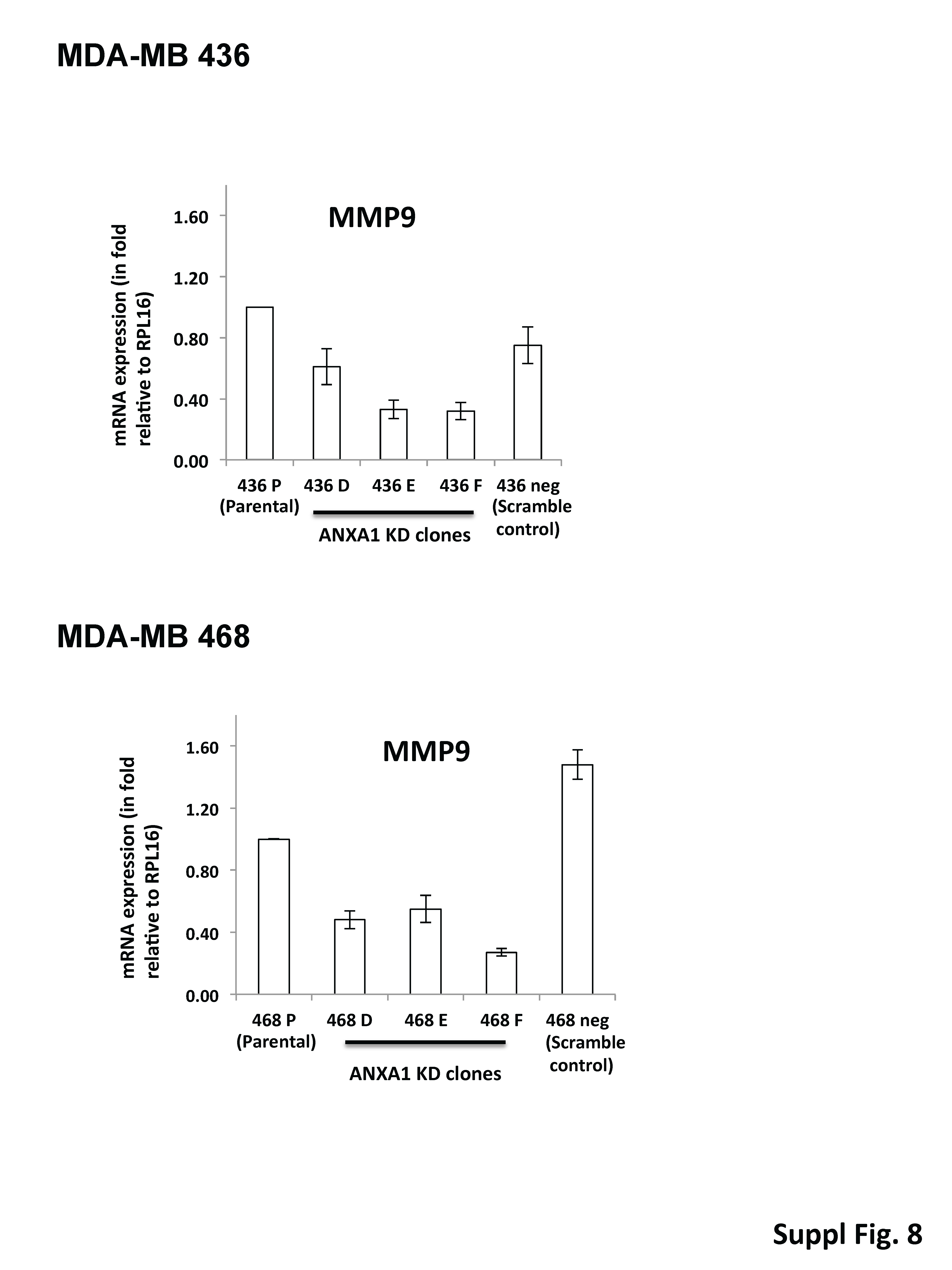

Supplement: S8 Fig — mRNA levels of MMP9 measured by QPCR in wild type and annexin A1 depleted clones of MDA-MB436 and MDA-MB-468 cells. All annexin A1 depleted clones were significantly different (p<0.05) relative to untreated/ parental cells. Experiments were repeated three times. (TIFF) [file pone.0127678.s008.tiff]
